# Relationship between neighborhood walkability and older adults’ physical activity: results from the Belgian Environmental Physical Activity Study in Seniors (BEPAS Seniors)

**DOI:** 10.1186/s12966-014-0110-3

**Published:** 2014-08-23

**Authors:** Veerle Van Holle, Jelle Van Cauwenberg, Delfien Van Dyck, Benedicte Deforche, Nico Van de Weghe, Ilse De Bourdeaudhuij

**Affiliations:** Department of Movement and Sport Sciences, Ghent University, Watersportlaan 2, B-9000 Ghent, Belgium; Department of Human Biometry and Biomechanics, University of Brussels, Pleinlaan 2, B-1050 Brussels, Belgium; Department of Geography, Ghent University, Krijgslaan 281 (S8), B-9000 Ghent, Belgium; Research Foundation Flanders (FWO), Brussels, Belgium

**Keywords:** Elderly, Built environment, Socio-economic status, Transport-related walking, Belgium

## Abstract

**Background:**

Adequate knowledge on environmental correlates of physical activity (PA) in older adults is needed to develop effective health promotion initiatives. However, research in this age group is scarce and most existing studies were conducted in North America. The present study aimed to examine relationships between GIS-based neighborhood walkability and objective and self-reported PA in community-dwelling Belgian older adults. Furthermore, moderating effects of neighborhood income levels were investigated.

**Methods:**

The Belgian Environmental Physical Activity Study (BEPAS) for Seniors is a cross-sectional study in older adults (≥65 yrs) and was conducted between October 2010 and September 2012. Data from 438 older adults living in 20 neighborhoods across Ghent (Belgium) were analyzed. Stratification of selected neighborhoods was based upon objective walkability and neighborhood income. Participants wore an accelerometer during seven consecutive days to obtain objective levels of moderate-to-vigorous physical activity (MVPA). Self-reported levels of transportation walking/cycling and recreational walking/cycling were assessed using the International Physical Activity Questionnaire (long, last 7 days version) adapted for the elderly. Multi-level regression analyses were conducted.

**Results:**

Findings showed a positive relationship between neighborhood walkability and weekly minutes of older adults’ self-reported walking for transportation (B = 4.63 ± 1.05;p < 0.001) and a negative relationship between walkability and accelerometer-derived low-light PA (B = −1.38 ± 0.62;p = 0.025). Walkability was not related to any measure of recreational PA. A walkability x income interaction was found for accelerometer-derived MVPA (B = -1.826 ± 1.03;p = 0.075), showing only a positive association between walkability and MVPA in low-income neighborhood residents.

**Conclusions:**

This was the first European study to examine walkability-PA relationships in older adults. These Belgian findings suggest that a high neighborhood walkability relates to higher levels of older adults’ transport-related walking. As transport-related walking is an accessible activity for older adults and easy to integrate in their daily routine, policy makers and health promoters are advised to provide sufficient destinations and pedestrian-friendly facilities in the close vicinity of older adults’ residences, so short trips can be made by foot. Neighborhood income moderated the relationship between walkability and objectively-measured MVPA. Increasing total MVPA levels in older adults should be a key topic in development of promotion initiatives and special attention should be paid to low-income neighborhood residents.

## Introduction

In the forthcoming decades, the global population of older adults (≥65 years) is expected to rise substantially [1]. As older age is often associated with physical frailty and functional limitations [2], aging will also affect the economic and health care sector through the rise in institutionalized individuals. Therefore, sustaining good physical functioning in older adults and consequently prolong independent community-based living is essential. Regular physical activity (PA) contributes to several beneficial health effects in all age groups [3–5], and may positively affect physical functioning, also in the elderly [6,7]. However, globally, 60-70% of older adults do not reach the recommended 150 weekly minutes of moderate-to-vigorous PA (MVPA) to achieve such health benefits [8], which highlights the need to promote PA in this age group. In order to create effective PA promotion initiatives in older adults, it is necessary to identify key PA correlates that specifically apply to this age group. During the past 15 years, researchers have adopted social ecological models to explain PA, which emphasize the importance of the built environment [9,10].

To date, research has identified several built environmental correlates of PA, including walkability of the residential neighborhood. Walkability reflects the built environments’ convenience for walking (primarily for transportation purposes; [11]) and is expressed as an index based on three key components; street connectivity, residential density, and land use mix diversity. Environments characterized by many interconnected streets, a high residential density and a mixture of different land uses (e.g., residential, commercial, institutional) are considered highly walkable. Previous research in adult populations (18-65 y) has shown consistent positive relationships between neighborhood walkability and several types of PA, in particular transport-related walking [12–14]. In elderly populations, walkability-PA relationships might be even stronger [15]. Older adults may experience more difficulties performing daily life activities as they age [16] and, hence, people with lower physical functioning may be more vulnerable to an unfavorable (less walking-friendly) environment than their functionally fitter peers [17,18]. However, few studies have examined the specific association between walkability and older adults’ health-enhancing PA, and those that did showed inconsistent results. Positive cross-sectional associations with neighborhood walkability were observed for US older adults’ MVPA [19,20], and for total walking in US older women [21] and older adults living in Iran [22]. Other research in older adults could not identify any relationships between walkability and older adults’ total PA [21,23,24] or measures of MVPA in older US women [21]. Regarding domain-specific PA, walkability was positively associated with older adults’ walking for transportation in the US [19,23], and their leisure-time walking [20]. However, in other studies, no significant relationships between walkability and older adults’ recreational PA were found [19,25,26]. These inconclusive results suggest that more research in older adults is needed before definite conclusions on the walkability-PA association for this age group can be drawn. Besides, no studies have examined the relationship between neighborhood walkability and older adults’ levels of low-intensity PA (LPA), even though this is the most prevalent type of PA in this age group [27–29], and recent research has identified positive relationships between older adults’ objectively-measured LPA and self-reported psychosocial and physical health factors [29]. Moreover, except for one study carried out in Iran, all above-mentioned studies were conducted in US or Australian settings. Study results from other continents such as Europe are currently lacking. North American and Australian study results may not be applicable to other geographical settings, as large differences in built environmental design exist between countries [30]. Research in adults has suggested that certain PA-environment relationships can be continent-specific [14] and this may also apply to elderly populations. Furthermore, cycling is a more prevalent type of PA in Europe compared to other continents such as North America [31] and in European adults, convincing evidence for positive associations between walkability and transportation cycling has been found [14]. European studies in older adults may reveal similar relationships, but such studies are currently lacking.

Another gap in the literature is the scarcity of studies in older adults taking into account potential moderators on relationships between walkability and PA levels. Health status and health behaviors such as PA may vary according to neighborhood income, a proxy for socio-economic status (SES) [32,33]. Research in adults on the moderating effects of SES on the walkability-PA association has found mixed results [34–36]. At present, no moderating effects of neighborhood income have been observed in walkability studies among US and Iranian older adults [19,22], but more research is needed to see whether or not this applies to older populations living in other countries as well.

In summary, the role of walkability as a PA correlate in older adults has been understudied, especially in countries other than North America and Australia. Moreover, research investigating the moderating effects of neighborhood income on walkability-PA relationships in older adults is scarce. Therefore, the first aim of the present study was to investigate the relationship between neighborhood walkability and older adults’ objective and self-reported PA levels. A second aim was to examine the moderating effect of neighborhood income on these relationships.

## Methods

### Design and setting

This study presents cross-sectional results of the Belgian Environmental Physical Activity Study in Seniors (BEPAS Seniors) among community-dwelling older adults (≥65 yrs). Measurements were conducted between October 2010 and September 2012 in Ghent, a medium-sized city (247,941 inhabitants in 2012; [37]) situated in the North of Belgium. The study protocol was approved by the Ethics Committee of the Ghent University Hospital.

### Neighborhood selection

Study participants were recruited across 20 neighborhoods in Ghent and its suburbs, which were selected from the 201 existing statistical sectors. Such sectors are the smallest units for which information on socio-demographics is available in Belgium. Each statistical sector approximately contains 1,000 inhabitants and neighborhoods comprised clusters from 1 to 5 adjacent sectors [35]. Neighborhoods were selected in two consecutive steps. First, statistical sectors were stratified according to objective walkability quartiles, determined using Geographical Information System (GIS) data. The walkability index applied for the present study was calculated with data on residential density, street connectivity and land use mix diversity, for which the calculation protocol has been described in detail by Van Dyck et al. [35,38]. Only neighborhoods in the top and bottom walkability quartiles were selected, representing high and low walkability, respectively. The average neighborhood size was 0.3 ± 0.1 km^2^ for high-walkable neighborhoods and 2.1 ± 2.1 km^2^ for low-walkable neighborhoods. In a second step of neighborhood selection, high and low-walkable neighborhoods were matched on high versus low neighborhood median annual household income data, provided by the Belgian National Institute of Statistics [35,39]. This matching resulted in the selection of four neighborhood types: high income/high walkability, high income/low walkability, low income/high walkability, and low income/low walkability [35,40].

### Participant recruitment

After neighborhood selection, addresses of older adults living in these neighborhoods were obtained through the Public Service of Ghent and a systematic random sample of 1750 community-dwelling older adults (≥65 y), stratified by gender and age (<75 y versus ≥75 y), was drawn. Selected older adults were contacted by postal mail through an information letter, in which the purpose of the study was explained and a visit of a trained interviewer during the subsequent two weeks was announced. Approximately one week after sending the letters, all selected older adults were visited at home. In case of absence at the moment of visit, up to two additional visit attempts were made on different days and different times of the day. Inclusion criteria for the study were: participants had to understand and speak Dutch, live independently (non-institutionalized) and be able to walk a couple of hundred meters without severe physical restrictions. In total, 1260 older adults were found at home when the trained interviewer visited them, of which 508 participated in the study. Six hundred twenty-seven (49.8%) people were not willing to participate and 125 (9.9%) were classified as “not eligible for participation due to severe physical restrictions”, which resulted in a response rate of 44.8% (508/1135 eligible participants found at home).

### Procedures

Participants were visited twice by the trained interviewer, with a mean interval of nine days in between two visits. During the first visit, respondents gave written consent for participation in the study and participated in a face-to-face interview, targeting perceived health status and their PA levels in the preceding week. Furthermore, the trained interviewer instructed participants how to wear an ActiGraph GT3X(+) accelerometer for measuring objective PA, which participants wore on waking hours during the subsequent seven consecutive days. The second home visit consisted of collecting the accelerometer and assessment of a socio-demographics questionnaire.

### Measures

#### Socio-demographics and self-reported physical functioning

Participants reported their age, living situation, educational level and their (former) occupation. Living situation was reported as being married; widowed; divorced; single and never had a partner; living together with a partner. Responses were collapsed into two categories “living with a partner” and “living without a partner”. Educational level was assessed by asking participants to report their highest educational degree according to following options: primary; vocational secondary; technical secondary; general secondary; college; university. Responses were dichotomized into “tertiary education” (comprising college and university) and “non-tertiary education” (all other options). Occupation was assessed through the question “What is/was your main occupation?”. Respondents were prompted to report the profession they performed for the longest period, choosing from household; education/teaching; employee; executive staff member; self-employed; profession; workman. Responses were categorized into “household”, “blue collar” (including workman and self-employed), and “white collar” (all other response options).

Self-reported physical functioning was measured with the physical functioning subscale of the Short Form 36 item Survey (SF-36; [41]). This subscale comprises ten activities and participants were asked to report on a 3-point scale whether or not they were restricted by their physical health to perform these activities (severely limited; somewhat limited; not limited). Activities included vigorous activities; moderate activities; lift or carry groceries; climb several flights; climb one flight; bend or kneel; walk a mile; walk several blocks; walk one block; bathe or get dressed. Subsequently, activities in which participants reported to be severely or somewhat limited were summed and this measure was dichotomized at its median value (2 limitations), yielding the categories “functionally limited” (≥2 limitations) and “not functionally limited” (<2 limitations).

#### Objective accelerometer-based PA

MVPA was objectively assessed with ActiGraph GT3X and GT3X + accelerometers (ActiGraph, Fort Walton Beach, FL, USA), which are valid and reliable tools to measure PA levels, also in older adults [42–44]. Accelerometers were attached to an adjustable elastic waist belt and worn above the right hip bone for at least five, to preferably seven consecutive days. Data were collected using 60-second epochs, according to the recommendations for accelerometer use in older adults [45]. Only data capturing the vertical plane were included for analysis in the present study. After data collection, raw accelerometer data were downloaded and exported to CSV files with the Actilife 6.0 software (Actigraph, Fort Walton Beach, FL, USA), which were subsequently screened, cleaned and scored using MeterPlus 4.3 (Santech, Inc.; [46]). A valid day was defined as a minimum of 10 wearing hours and participants with less than five valid days of data were excluded for analysis. Periods covering ≥ 90 minutes of consecutive zeros were categorized as “non-wearing” [47]. Activity categories were defined with the Freedson et al. [48] cut points. Registrations below 101 counts.min^−1^ were categorized as sedentary behavior, 101 through 1,951 counts.min^−1^ were considered light-intensity PA (LPA), and all accelerometer counts above 1,951 counts.min^−1^ were defined as moderate-to-vigorous PA (MVPA). Analogous to the study in US older adults of Buman et al. [29], LPA was additionally subdivided into low-light and high-light PA according to the elderly-specific Copeland & Esliger [43] threshold of 1,041 counts.min^−1^. “Low-light” intensity may refer to standing activities, such as ironing or doing the dishes, or to very light household chores, such as tidying up the table. The “high-light” intensity category, on the other hand, reflects the transitional zone at the higher end of the LPA and the lower end of the MVPA continuum. This category can include activities such as walking at < 4 km.hour^−1^, or doing household chores such as vacuuming and hanging out the washing. For the present study, activity counts were converted into weekly minutes of low-light PA, high-light PA, and MVPA and used as dependent variables in the analyses.

#### Self-reported transport-related and recreational PA

Self-reported PA levels were assessed with the long International Physical Activity Questionnaire (IPAQ; www.ipaq.ki.se, last 7 days interview version), comprising questions on transportation and recreational PA. Elderly-specific reliability of IPAQ for separate PA domains was tested in a previous study (*Van Holle et al., submitted*), suggesting acceptable seven days test-retest results. Within a time frame of the last seven days, participants reported the frequency (number of days) and the average time per day (hours and minutes) spent doing transport-related PA (walking and cycling reported separately) and recreational activity (walking, cycling and other MVPA for recreational purposes reported separately). Interviewers prompted participants to report only those activities with a minimum duration of 10 consecutive minutes. Weekly minutes of time spent doing each activity were calculated by recoding the reported average daily time to minutes and multiplying this with the reported number of days, resulting in the outcome variables “transport-related walking”; “transport-related cycling”; “recreational walking”; “recreational cycling”; and “other recreational MVPA”. All of these variables were truncated to a maximum of 840 weekly minutes (~2 hours per day).

#### Statistical analyses

SPSS 20.0 (SPPS Inc., Chicago, IL, USA) was used to calculate descriptive statistics. Because some of the objective and perceived PA variables were positively skewed, all PA variables were square root transformed to improve normality. Transformed variables were used in all analyses reported below, except for analyzing descriptive statistics, which were calculated with the raw data (Table 1). MLwiN 2.25 multilevel linear regression modeling (two levels: neighborhood-participant) was applied to examine the main and the interaction effects of neighborhood walkability (high vs. low) and income (high vs. low) with each PA measure. All analyses were controlled for gender, educational level, living situation, age and physical functioning, as these variables were significantly related to at least one of the PA variables. Because regression coefficients represented relationships with square root transformed PA variables, for each regression analysis, predicted weekly minutes of PA for the each type of neighborhood were calculated and squared. The predicted values were calculated with all covariates fixed at their mean. The predictions were obtained from MLwiN’s customized prediction window [49] and predicted values are reported in the Results section to facilitate interpretation of the observed relationships. Statistical significance was set at p < 0.05 for interpreting main effects. Significance levels for interpreting interactions were set at p < 0.1 [50].

**Table 1:** **Sample characteristics and levels of PA for the total sample (n = 438) and stratified by neighborhood type**

	**Total**	**High walkability**			**Low walkability**		
		**TOTAL**	**HIGH INCOME**	**LOW INCOME**	**TOTAL**	**HIGH INCOME**	**LOW INCOME**
*Personal factors*							
Gender (% female)	54.1	55.6	55.1	56.0	52.6	50.9	54.1
Age in years (M ± SD)	74.3 ± 6.2	74.2 ± 6.6	74.5 ± 6.6	73.9 ± 6.7	74.5 ± 5.9	73.9 ± 5.3	75.1 ± 6.1
Living situation (%partner)	65.8	61.1	68.2	54.4	70.7	75.5	66.1
Educational level (%tertiary)	38.4	48.2	53.8	43.0	28.4	38.7	18.3
Former occupation (%)							
household	18.2	15.5	11.2	19.5	20.9	18.9	22.9
blue collar	27.1	21.5	21.5	21.2	33.1	27.4	38.5
white collar	54.7	63.2	67.3	59.3	46.0	53.7	38.6
Phys. Functioning (%not limited)	34.7	35.4	32.7	37.9	34.0	38.7	29.4
*Accelerometer PA (min.wk* ^*−1*^ *)*							
Low-light PA (M ± SD)	1577.1 ± 485.3	1528.8 ± 493.5	1596.4 ± 491.6	1466.3 ± 489.1	1627.2 ± 472.5	1722.9 ± 420.4	1534.2 ± 502.8
High-light PA (M ± SD)	213.2 ± 162.0	207.6 ± 157.0	208.7 ± 143.2	206.6 ± 169.4	218.9 ± 167.2	239.6 ± 162.4	198.8 ± 170.2
MVPA (M ± SD)	110.5 ± 116.8	129.8 ± 130.4	115.0 ± 119.7	143.5 ± 138.8	90.4 ± 97.0	102.6 ± 104.8	78.4 ± 87.5
*Self-reported PA (min.wk* ^*−1*^ *)*							
Transportation walking (M ± SD)	86.1 ± 140.9	128.2 ± 166.9	95.3 ± 142.4	158.6 ± 182.0	42.3 ± 88.6	43.0 ± 103.1	41.7 ± 72.2
Transportation cycling (M ± SD)	36.6 ± 96.9	37.5 ± 97.7	37.8 ± 90.4	37.2 ± 104.4	35.6 ± 96.2	40.4 ± 119.5	30.8 ± 66.5
Recreational walking (M ± SD)	83.0 ± 159.1	88.7 ± 163.4	89.2 ± 156.2	88.3 ± 170.5	77.1 ± 154.7	63.3 ± 131.3	90.5 ± 174.1
Recreational cycling (M ± SD)	39.9 ± 123.5	35.0 ± 120.1	38.6 ± 128.8	31.8 ± 111.9	45.0 ± 131.0	62.3 ± 166.9	28.2 ± 79.7
Other recreational MVPA (M ± SD)	46.7 ± 122.9	41.7 ± 106.8	30.5 ± 87.3	52.0 ± 121.5	51.8 ± 137.8	30.8 ± 92.0	72.2 ± 168.9

Note: Descriptives of all PA variables show means and standard deviations of the raw data.

M = mean; SD = standard deviation; PA = physical activity; MVPA = moderate-to-vigorous PA.

## Results

### Sample characteristics and PA levels

Descriptive characteristics of the total sample, as well as those stratified for walkability and neighborhood type, are displayed in Table 1. In total, 438 participants with a mean age of 74 ± 6 years (range 65-92 y) provided complete data and were included for analysis. The majority of the sample was lower educated, had performed a white-collar job and reported to be functionally limited in at least two daily life activities. Fifty-four percent of participants were women, which is similar to the gender distribution in Belgium (54% women; [39]). In contrast, compared to the Belgian population of older adults, a higher percentage of our participants lived with a partner (65.8% versus 56.2% for Belgium; [39]).

### Main effects of neighborhood walkability

Table 2 presents the main effects of neighborhood walkability on older adults’ levels of PA. Residents of high-walkable neighborhoods self-reported more weekly minutes of transport-related walking (predicted value 76.0 min.wk^−1^) than residents of low-walkable neighborhoods (16.7 min.wk^−1^; p < 0.001). On the other hand, older adults living in low-walkable neighborhoods engaged in more weekly minutes of objectively-measured low-light PA than their peers living in high-walkable neighborhoods (1586.0 min.wk^−1^ vs. 1478.4 min.wk^−1^; p = 0.025). Walkability was unrelated to older adults’ objectively-measured high-light PA, self-reported transportation cycling and all measures of self-reported recreational PA.

**Table 2:** **Associations (B, 95% and 90% CI) between neighborhood factors and older adults’ PA (n = 438)**

	**Walkability**		**Income**		**Walkability x income**	
	B	95% CI	B	95% CI	B	90% CI
*Accelerometer PA*						
Low-light PA	−1.382*	−2.589; −0.175	1.855*	0.765; 2.945	−0.318	−2.118; 1.482
High-light PA	−0.495	−1.426; 0.436	0.534	−0.387; 1.455	−0.796	−2.324; 0.732
MVPA	2.057*	0.675; 3.439	0.409	−1.004; 1.822	−1.826^¥^	−3.516; −0.134
*Self-reported PA*						
Transport-related walking	4.625**	2.571; 6.679	−2.144	−4.923; 0.635	−2.202	−5.063; 0.659
Transport-related cycling	0.179	−1.682; 1.168	0.160	−0.896; 1.216	0.883	−0.833; 2.599
Recreational walking	0.256	−1.328; 1.840	−0.418	−1.955; 1.119	1.293	−1.228; 3.814
Recreational cycling	−0.349	−1.764; 6.679	0.753	−0.631; 2.167	−0.587	−2.854; 1.680
Other recreational MVPA	0.042	−1.438; 1.522	−1.651*	−2.807; −0.495	0.797	−1.105; 2.699

All PA variables are expressed as square root transformed min.wk^−1^.

**p < 0.001; *p < 0.05; ^¥^p < 0.10.

Analyses are controlled for gender, age, living situation, educational level and physical functioning.

### Main and moderating effects of neighborhood income

Table 2 further presents the associations between neighborhood income and participants’ PA levels. Older adults living in high-income neighborhoods had higher levels of objectively-measured low-light PA than their peers living in low-income neighborhoods (1610.4 min.wk^−1^ vs. 1463.9 min.wk^−1^ p = 0.009), but lower levels of self-reported other recreational MVPA (4.5 min.wk^−1^ vs. 14.4 min.wk^−1^; p = 0.014). Neighborhood income was unrelated to all other PA variables. A walkability x income interaction was found for accelerometer-derived MVPA (B = -1.826 ± 1.03; p = 0.075), showing only a positive association between walkability and MVPA in residents of low-income neighborhoods, and not in those living in high-income neighborhoods. Residents of high walkability/low income neighborhoods accumulated 106.0 weekly minutes of objectively-measured MVPA, whereas older adults living in low walkability/low income neighborhoods accumulated 67.8 weekly minutes of MVPA (p = 0.023). No other walkability x income interactions were observed.

## Discussion

Our study was the first to examine associations between neighborhood walkability and different types of PA in European older adults. Previous research on these relationships was predominantly conducted in North America, where the built environment can differ substantially from other continents [30]. Consequently, this raised questions on the applicability of North American results to other geographical contexts and, therefore, the present study sought to reveal relationships in a Western European context.

A first aim of our study was to identify main effects of walkability in relation to older adults’ objectively-measured and self-reported PA. Regarding objective levels of PA, the present study investigated associations between walkability and LPA in addition to MVPA, given that the vast majority of older adults’ daily total PA consists of low-intensity activities [27,28]. Accelerometer low-light PA was the most prevalent type of all objectively-measured PA and there was a negative association with neighborhood walkability. Presumably, residents of low-walkable neighborhoods spend more time doing activities indoors, such as cleaning up or doing other small household chores (e.g., ironing). House-based activities may be less physically challenging and of shorter duration than outdoor activities (e.g., walking to a shop). In contrast to the significant results found for low-light PA, walkability was unassociated with older adults’ high-light PA. A univocal explanation for this finding is difficult to formulate, as no other studies have investigated relationships between walkability and older adults’ accelerometer LPA yet and results cannot be compared. Nevertheless, these contrasting findings for low-light versus high-light PA indicate that splitting total LPA into low-light and high-light PA, according to the Copeland cut point of 1,041 counts.min^−1^ [43] may be useful to get a better insight into how the built environment is related to older adults’ different types of LPA.

Next to objectively-measured PA, we examined self-reports of older adults’ domain-specific PA. Walkability was positively associated with older adults’ walking for transportation, and this finding supports results from Belgian Environmental Physical Activity Study in adults (BEPAS Adults) [35], as well as other non-European research among adults [19,23,25] and older adults [19,23]. As walking from place to place is an accessible and low-cost activity, which older adults can easily integrate into their daily routine, this positive relationship is very promising from a health promotion perspective. Given that 65% of the participants reported to have at least two physical limitations that restricted them from doing daily-life activities, short trips may be most desirable. Access to destinations (e.g., shops, public services, places for social interaction) in the close vicinity of the home residence may thus be a key environmental correlate behind this significant relationship. Previous qualitative [51,52] and quantitative [53–55] research on the PA-environment relationship in older adults has already provided some evidence for the importance of destinations for walking in the elderly. However, having destinations nearby only relates to one of the three components of walkability (i.e., land use mix diversity), whereas the walkability index also includes street connectivity and residential density. Both of these factors have been positively associated with older adults’ transport-related walking in some prior studies [15,56]. With regard to our findings, a higher street connectivity may facilitate walking because street segment length and block size are smaller and destinations are more directly accessible [56,57]. Walkability was not associated with any measure of self-reported recreational PA in our study. This contrasts with the BEPAS Adults, where participants walked more for recreation in a high-walkable neighborhood [35], but is consistent with some previous work in older adults [19,25,26]. Perhaps, other aspects of the physical environment could be more important in explaining recreational PA among older adults, such as the presence of parks or traffic levels [58]. Another possibility is that socio-ecological factors other than the physical environment, for instance social support or strength of one’s social network [20,59], may be more closely related to recreational PA in this age group.

The present study was the first to examine associations between walkability and cycling in the elderly, but no significant relationships were found. This might be due to a low prevalence of cycling in older adults. As cycling requires a certain basic fitness and balance/agility level, participants with physical limitations may have perceived themselves as more vulnerable to get injured. Another reason for the lack of association may be that older adults who did cycle, cycled longer distances, beyond the boundaries of their residential neighborhood where walkability was measured.

A second aim of our study was to investigate possible moderating effects of neighborhood income (a proxy for SES) on the walkability-PA relationships. In the present study, older adults living in low-income neighborhoods accumulated about 11 additional weekly minutes of objectively-measured MVPA when they resided in a high-walkable, compared to a low-walkable neighborhood. In contrast with prior studies among Iranian and US older adults, which could not identify any income x walkability interactions [19,22], this was the first study to show that the observed relationship between walkability and PA was only present among older adults living in low-income neighborhoods. Our observed interaction indicates that in Belgium, the built environment may be of greater importance for certain population subgroups and public health promotion initiatives should be adapted accordingly. Economic factors might partly explain this finding; low-income neighborhood residents may have less access to the private car (i.e., own a car themselves or have significant others owning a car) than residents of high-income neighborhoods and may therefore spend more time in their neighborhoods, making them more dependent of walkability of their residential environment. In contrast, high-income neighborhood residents might be more mobile and could spend more time outside the neighborhood boundaries, which in turn could explain why walkability of their residential neighborhood was unassociated with MVPA levels in this group of older adults.

### Strengths and limitations

A first strength of BEPAS Seniors is that it is the first European study examining specific walkability-PA relationships in older adults and to our knowledge, the only study investigating associations with cycling and light-intensity PA. Hence, our results are useful in updating the current knowledge on the PA-environment relationship in older adults across the broad spectrum of geographical settings. Secondly, the study design of BEPAS Seniors is identical to the design of BEPAS Adults [35], making it possible to get a better insight in environment-PA relationships across different age groups and identify age-specific correlates of Belgians’ PA levels. Besides, the study design is similar to the US Neighborhood Quality of Life Study for Seniors [15,19,23], providing the opportunity to compare study findings cross-nationally and even cross-continentally. A third strength of the present study is that we assessed domain-specific measures of self-reported PA. Our different outcomes regarding transport-related versus recreational PA confirm that the PA-environment relationship can be domain-specific [12,60]. Moreover, self-reported PA levels were collected through face-to-face interviews. As older adults may experience more cognitive difficulties when responding to a questionnaire [61], the guidance by the interviewer was experienced to be beneficial for this population of elders because more accurate responses can be obtained. In addition, as a previous study suggested that older adults tend to over-report levels of total MVPA (Van Holle et al., submitted), accelerometers were used to objectively measure levels of total MVPA in the present study, which is another strength of the study. However, some limitations need to be acknowledged as well. First, our study only presents cross-sectional data and relationships cannot be assumed to be causal. Longitudinal studies in older adults are recommended to determine whether the identified relationships change or last over time. Additionally, using longitudinal designs will enable researchers to examine whether a more activity-friendly built environment (e.g., higher walkability) may help older adults to maintain their PA levels over time. Moreover, natural experiments are encouraged, to investigate if over-time structural changes in the built environment will cause changes in older adults’ PA. A second limitation is that no information on the specific context of PA was available, so we cannot make sure that the objectively-measured and self-reported PA occurred in the residential neighborhood. Future studies incorporating GPS measurements may provide such additional information.

## Conclusions

In conclusion, objective neighborhood walkability was negatively associated with accelerometer-derived weekly minutes of low-light PA and positively associated with self-reported weekly minutes of transportation walking among Belgian older adults. Walkability was unrelated to cycling, nor to any measure of recreational PA. These results suggest that in older adults, neighborhood walkability predominantly applies to the transport-related PA domain, and walking in particular. As walking for transportation is an accessible activity for older adults, which is easy to integrate in their daily routine, these results are very valuable from a health promotion perspective. Policy makers and health promoters are advised to provide sufficient destinations in the close vicinity of older adults’ residences, in order to facilitate short utilitarian walking trips. Moreover, walking could be facilitated through optimization of street connectivity (e.g., small block sizes). Results of the present study further showed that neighborhood income levels moderated the walkability-MVPA relationship, and suggest that improving neighborhood walkability could be an effective approach to facilitate PA among older adults living in lower SES areas. Increasing total MVPA levels in older adults is highly relevant, especially for residents of low-income neighborhoods. In summary, some, but not all results were consistent with earlier research among older adults in other geographical areas such as the US. As this was the first European study to examine specific walkability-PA relationships in this age group, our findings are very useful for updating the current knowledge and may indicate a need for area-specific health promotion initiatives across different geographical contexts.

## Abbreviations

PA: Physical activity
MVPA: Moderate-to-vigorous-intensity physical activity
LPA: Light-intensity physical activity
GIS: Geographical Information System
